# The Imbalance of Mitochondrial Homeostasis of Peripheral Blood-Derived Macrophages Mediated by MAFLD May Impair the Walking Ability of Elderly Patients with Osteopenia

**DOI:** 10.1155/2022/5210870

**Published:** 2022-03-24

**Authors:** Xiaojun Wang, Xuanqi Liu, Peqing He, Kangwei Guan, Yijing Yang, Yiming Lei, Jianhua Cai, Wenhao Wang, Tao Wu

**Affiliations:** ^1^Department of Traditional Chinese Medicine, Huadong Hospital Affiliated to Fudan University, Shanghai 200040, China; ^2^Shanghai Key Laboratory of Clinical Geriatric Medicine, Huadong Hospital Affiliated to Fudan University, Shanghai 200040, China; ^3^Department of Respiratory and Critical Care Medicine, Huadong Hospital Affiliated to Fudan University, Shanghai 200040, China; ^4^Department of General Surgery, Huadong Hospital Affiliated to Fudan University, Shanghai 200040, China

## Abstract

**Introduction:**

Many Asian cohort studies have shown that nonalcoholic fatty liver disease (NAFLD), now renamed as metabolic dysfunction-associated fatty liver disease (MAFLD), increases the risk of osteoporosis, yet the effect of MAFLD on elderly patients with osteopenia (OPe) has not been reported.

**Objective:**

This study aimed to explore the influence of MAFLD on the function of macrophages in patients with OPe.

**Methods:**

A total of 107 elderly OPe patients with or without MAFLD who visited the Huadong Hospital Affiliated to Fudan University (Shanghai, China) between January 1st, 2021, and September 30th, 2021, were evaluated for an interviewer-assisted questionnaire, as well as clinical and biological assessments.

**Results:**

Comparing two groups of elderly patients with the same bone mass level, we found that the six-minute walking distance (*P* = 0.012) and short physical performance battery (SPPB) score (*P* = 0.0029) of the elderly OPe patients with MAFLD are worse than those in OPe patients without MAFLD. Our results confirmed that the mitochondrial reactive oxygen species (mtROS) in peripheral blood of OPe patients with MAFLD was significantly higher than those without. We also observed the mitochondrial metabolism level of peripheral blood-derived macrophages in the included patients and peripheral blood macrophages in patients with MAFLD with more unbalanced mitochondrial dynamics of macrophages, more weakened mitochondrial respiratory capacity, and greater mitochondrial microstructure damage, when compared with the elderly patients without MAFLD.

**Conclusions:**

To conclude, our data revealed that MAFLD itself may aggravate the inflammatory state in elderly OPe people due to mitochondrial homeostasis imbalance of peripheral blood macrophages. Damaged monocyte-macrophages might trigger attenuation of the walking ability of OPe patients.

## 1. Introduction

Metabolic dysfunction-associated fatty liver disease (MAFLD), formerly known as nonalcoholic fatty liver disease (NAFLD), due to its high global prevalence, causes a huge economic burden to society [1]. Unhealthy habits such as sedentary and less active lifestyles and unhealthy dietary patterns are closely related to a high incidence rate of MAFLD [2], which can be diagnosed accurately by certain evaluating criteria like obesity, type 2 diabetes mellitus (T2DM), or metabolic disorders [3]. The prevalence of fatty liver disease (FLD) and osteoporosis is increasing in elderly people [4, 5]. Therefore, it has also been studied in various Asian cohorts reporting that FLD increases the risk of osteoporosis in the elderly [6, 7], though the association between FLD and osteoporosis remains ambiguous [8, 9].

Osteopenia (OPe) refers to the decrease in bone mass per unit volume of bone, which can manifest into osteoporosis on further aggravation. In the elderly population, fractures can occur as a primary clinical endpoint for both osteoporosis and persistent history of falls [10] due to a gradual decrease of lower limb muscle strength, which directly affects the balancing function and the walking ability [11]. Fatty liver can increase the risk of fractures in osteoporotic patients [12] and may interact through complex pathways such as inflammatory mediators, hormones, substance metabolism, and intestinal flora imbalance in conjunction with osteoporosis [8]; however, it has not been clarified yet.

The excessive triglycerides accumulation in the liver during MAFLD might lead to pro-inflammatory and anti-inflammatory factor imbalance, affecting the function of immune cells whose homeostasis is largely influenced by their metabolic activities [13]. Thus, specific metabolic adaptability should be acquired to support their diverse immune functions [14]. As an important member of innate immunity, the function of macrophages is strictly regulated by metabolic pathways and metabolic intermediates [15, 16], which sometimes get altered by innate and acquired immunity changes, known as “inflammatory aging” due to chronic low-level systemic inflammation that can affect macrophage polarization [17, 18]. This study attempts to investigate whether MAFLD will affect the function of macrophages in patients with OPe, resulting in the decrease of walking and balance ability to aggravate the risk of fracture or fall in elderly patients.

## 2. Methods

### 2.1. Data Extract and Bioinformatics Analysis

The transcription and expression profiles by an array of 28 elderly patients with osteoporosis and 73 females with low or high bone mass were downloaded from the ArrayExpress and Gene Expression Omnibus (GEO) databases, which included 28 bone tissue (E-MEXP-1618) and 73 circulating monocytes (Query DataSets for GSE56816). The disease-associated targets were based on Gene disease database (DisGeNET) (http://www.disgenet.org) and ImmPort (The Immunology Database and Analysis Portal). By using the DAVID 6.8 database [19], the Kyoto Encyclopedia of Genes and Genomes (KEGG) signal pathway enrichment of the important target was analyzed. Gene Oncology (GO) annotation was carried out using clusterProfiler, and R Package was used for comparing biological themes among gene clusters [20].

### 2.2. Subjects and Study Design

The study protocol was reviewed and approved by the Institutional Ethics Committee of Shanghai Huadong Hospital (2019 K111, 2021 K073). All patients included in this study signed informed consent, and they were enrolled at Huadong Hospital in Shanghai, China, from January 1st, 2021, to September 30th, 2021. In total, 107 participants completed an interviewer-assisted questionnaire and muscle strength evaluation of the research center. At the same time, their biological information was collected. Patient exclusion criteria are given in the flowchart (Supplement 1).

### 2.3. Biochemical Determination

Blood samples were collected from each participant in the morning after 12 h of fasting, and assayed at the Huadong Hospital Laboratory. The Chronic Kidney Disease Epidemiology Collaboration equation was used to determine glomerular filtration rate (Estimated Glomerular Filtration Rate (eGFR); mL/min/1.73 m^2^) to assess renal function. In addition, triglycerides (TG), total cholesterol (TC), serum creatinine (Scr), serum uric acid (SUA), blood urea nitrogen (BUN), alanine aminotransferase (ALT), aspartate transaminase (AST), blood phosphorus and calcium (P and Ca), N-Propeptide of Type I Procollagen (P1NP), *β*-Crosslaps (CROSSL), parathyroid hormone (PTH), osteocalcin (OSTEOC), C reactive protein (CRP), and high- and low-density lipoprotein (HDL and LDL) levels in serum were measured.

### 2.4. Criteria for MAFLD and Human Liver Samples

MAFLD was diagnosed based on blood biomarkers and imaging evidence for hepatic fat deposition, besides the three other criteria, which included metabolic dysregulation, T2DM, and overweight [3]. The hepatic samples were collected from patients undergoing liver biopsies after approval from the Institutional Ethics Committee of Shanghai Huadong Hospital (2021 K073). Liver tissue samples were collected from six participants whose age was above 60 years old.

### 2.5. Bone Mineral Density (BMD) Measurement

According to specific instructions, we determined BMD of the total hip, femoral neck, and lumbar spine through single X-ray absorptiometry (HOLOGIC, Discovery W, USA). As per the guidelines of the World Health Organization (WHO), we considered a patient to be suffering from OPe when the BMD *T*-score ranged from −1.0 to −2.5 and osteoporosis when the *T*-score was <−2.5. Severe osteoporosis was indicated by BMD <–2.5, or brittle fractures [21].

### 2.6. Muscle Strength Measurement

To evaluate muscle strength, grip strength was measured by using an electronic hand dynamometer (CAMRY, MODEL: EH101, CHINA). We considered the highest value from three measurements on bilateral sides as the maximal grip strength. The five-time sit-to-stand test (FTSST) and six-minute walking distance were conducted to determine the strength of the lower extremity muscle. Typically, in the FTSST, the subjects were instructed to sit at the edge of a chair, fold their arms, and hit the chair with their buttocks in every repetition. The participants were given standardized instructions in a six-minute walking test and asked to walk “as far as possible in a 6-min period” along a 90 m course. The subjects were verbally encouraged at intervals of 30 s by standardized phrases and allowed to sit on chairs throughout the experimental period. Six minutes later, we determined the distance walked (to the nearest meter).

The short physical performance battery (SPPB) score, which consisted of 3 components, gait speed, repeated chair stands, and standing balance, is a subjective approach to determine the alteration of physical performance and balance ability among the elderly population as well as a standard measure both for research and clinical practice [22]. Additionally, an inextensible tape was used to flex the knees at 90°; then, we determined the average calf circumference at the broadest level of the bilateral calves in the relaxed and seated position.

### 2.7. Study Questionnaires and Clinical Assessments

The Pittsburgh Sleep Quality Index (PSQI) provided data regarding subjective sleep quality in the previous month. The nurses or assistant nurses were invited to assess the Mini Nutritional Assessment-Short Form (MNA-SF). We also determined the body weight (BW) and body height (BH) (with no shoes on), and then, the body weight (kg) was divided by the square of the body height (m^2^) to determine body mass index (BMI). We collected baseline data from all subjects, including age, gender, drinking history, smoking history, medical history, and the usual amount of exercise, by distributing the self-administered questionnaires. We measured the waistline, BH, and BW during the interview, and subjects were instructed to wear light clothing and no shoes. Blood samples were collected from participants who had fasted for 8 h in the morning and analyzed at the Huadong Hospital laboratory.

### 2.8. Isolation of Peripheral Blood Mononuclear Cells (PBMCs) and Magnetic Bead Sorting

Blood samples were collected from 20 OPe cases with MAFLD, 20 OPe patients without MAFLD, and 20 patients with severe osteoporosis. Then, we extracted total blood by Ficoll density gradient centrifugation to obtain PBMCs. CD14+ monocytes were magnetically sorted by the MojoSort™ Human Pan Monocyte Isolation kit (Biolegend, San Diego, CA, USA).

### 2.9. Cell Culture and Treatment

We cultured the isolated PBMCs and human myeloid leukemia mononuclear (THP-1) cells in Dulbecco's modified eagle medium (DMEM) with 20% fetal bovine serum (FBS; Millipore, USA) at 5% CO2 and 37°C. Additionally, we cultivated human hepatocellular carcinoma (HepG2) cells in DMEM low sugar medium that contained 5% FBS. To induce overloading of free fatty acids (FFAs), we cultured cells till they reached 70~80% confluence and exposed them to a mixture of 1 mM long-chain FFAs (palmitic acid and oleic acid (PAOA)) (oleic acid: palmitic acid =1 : 2) for 24 h [23].

### 2.10. Flow Cytometry (FCM) and Enzyme-Linked Immunosorbent Assay (ELISA)

FCM was used to examine the cells (FACS Aria ™ II, BD Bioscience, NJ, China). The Flowjo™ 10 software was used to analyze the data. Peripheral blood macrophages were defined as CD14+ and CD16+ cells, from which M1 and M2 macrophages were identified as CD86 + HLR-DR+ or CD163 + CD206+ cells, respectively, and monocytes were derived from the patients. All antibodies were obtained from Biolegend (San Diego, CA, USA). ELISA was used to determine interleukin-6 (IL-6) and IL-8 levels in the plasma (X-Y Biotechnology, Shanghai, China).

### 2.11. Reverse Transcription-Polymerase Chain Reaction (RT-PCR)

We used TRIzol reagent (Invitrogen, USA) to extract total cellular RNA, which was later used to prepare cDNA through reverse transcription using a kit (Vazyme Biotech, Nanjing, China). The prepared cDNA served as the template for RT-PCR that was performed using a Roche Light Cycler 96 system (Roche, Switzerland) with the 2X SYBR-Green-based qPCR reagent. The 2-*ΔΔ*Ct method was used to determine the relative gene expression [24], and GAPDH was used as the endogenous control. Each assay was performed three times. Supplement 3 lists the sequences of all the primers used.

### 2.12. Oxygen Consumption Rate (OCR) and Extracellular Acidification Rate (ECAR) Measurements

Glycolysis and mitochondrial respiration were quantified using the Seahorse XF Analyzer (Seahorse XF96, Agilent, USA). To measure OCR, we incubated cells (5 × 103/well) in 96-well XF96 plates overnight. The Seahorse Assay medium was used to substitute the original medium in the XF96 plates 1 h before measurement. We determined OCR under three conditions, which included baseline, 0.5 *μ*M antimycin and 0.5 *μ*M rotenone, and 0.3 *μ*M carbonyl cyanide p-(trifluoromethoxy) phenylhydrazone (FCCP) and 1 *μ*M oligomycin. To quantify ECAR, we injected the glycolysis inhibitor 2-deoxy-D-glucose (2-DG) to stop glycolytic acidification.

### 2.13. Mitochondrial Isolation and Reactive Oxygen Species (ROS), Mitochondrial Reactive Oxygen Species (mtROS) Quantification

The mitochondria were isolated from PBMCs using a Mitochondria Isolation Kit (Thermo Fisher Scientific). MitoSOX™ Red (Yeasen, China) and DCFH-DA (Beyotime, China) were used to determine mtROS and intracellular ROS contents according to certain protocols. The average fluorescence intensities of mtROS and intracellular ROS were determined using FCM (FACS Aria TM II, BD Bioscience, NJ, China). Transmission electron microscopy (TEM) (suht7700, Hitachi, Japan) was used to examine mitochondrial morphology which was used to examine mitochondrial ultrastructural damage by other researchers [25, 26].

### 2.14. Mitochondrial Membrane Potential (MMP) and Mitochondrial Permeability Transition Pore (MPTP) Assay

We determined MMP using the mitochondrial membrane potential detection kit (JC1, Beyotime Biotech, China) following specific protocols. Later, we tested the opening degree of the MPTP using the MPTP Detection Kit (Beyotime Biotech, China), following specific instructions (http://www.beyotime.com/index.htm). Flow cytometry was used to analyze the MMP and MPTP opened of the macrophages derived from peripheral blood [27–29].

### 2.15. Immunofluorescence (IF) and Nile Red Staining

The cells were fixed with paraformaldehyde (PFA) at room temperature (RT). Next, 10% BSA (Sangon Biotech, Shanghai, China) was used to block the cells, followed by incubation using primary antibodies (1 : 100, ab113748, Abcam) for 2 h at 37°C; the cells were then washed thrice with PBS. The cells were incubated with a secondary antibody (1 : 100, A-21244, Thermo Fisher Scientific) for 1 h at 37°C. Supplement 4 lists the antibodies used in this study. The instructions for the use of all antibodies can be found on the website (https://abclonal.com.cn,https://www.ptgcn.com/).

Nile Red (Sangon Biotech, Shanghai, China) (1 mM) was used to observe the intracellular lipid droplets. We mounted the cells with DAPI (abs9235, Absin) using a laser confocal cell culture dish (Thermo Fisher Scientific, NY, USA). A confocal imaging system (LSM 780) (Carl Zeiss, Jena, Germany) was used to take images.

### 2.16. Western Blotting (WB) Assay

We extracted total cellular or hepatic tissue proteins for the WB assay using the RIPA lysis buffer. Then, the bicinchoninic acid (BCA) Protein Assay Kit (Beyotime, China) was used to determine the protein content. Supplement 4 lists the antibodies used in this study. The instructions for the use of all antibodies can be found on the website (https://abclonal.com.cn,https://www.ptgcn.com/).

### 2.17. Oil-Red O Staining and Immunohistochemistry (IHC)

We used the Oil-Red O staining kit (Abcam, USA) for Oil-Red O staining as per specific protocols. The sections were also incubated with the silent mating type information regulation 1 (SIRT1) primary antibody (1 : 100, Abcam). Each tissue section was mixed with the reaction enhancer (Record Biological, Shanghai, China). The sections were then incubated with the enhanced enzyme-conjugated goat anti-rabbit IgG polymer (Record Biological, Shanghai, China). Images were captured using a light microscope (Nikon ECLIPSE 80i, Nikon, Japan).

### 2.18. Transfection with Small Interfering RNA (siRNA)

According to specific protocols, the siRNA was transfected into CD14+ PBMCs using the Lipofectamine RNAiMax reagent (100 nM, Thermo Fisher Scientific, USA). The siRNA sequence for SIRT1 was 5′ − GGCTGGTGATCGCAGATTT − 3′ (RiboBio, Guangzhou, China).

### 2.19. Statistical Analysis and Art Work

Differentially expressed genes are displayed in a heat map in R. All statistics were performed using Prism (version 8.0.2 for Mac; GraphPad Software, San Diego, CA). The use of 1-way ANOVA established statistical comparisons between the different groups. All values are presented as the mean ± SD *P* values were specified as follows: ∗*P* < 0.05; ∗∗*P* < 0.01; ∗∗∗*P* < 0.005; ∗∗∗∗*P* < 0.0001.

## 3. Results

### 3.1. Potential Ways of Interaction between Fatty Liver and OPe

**Figure 1:**
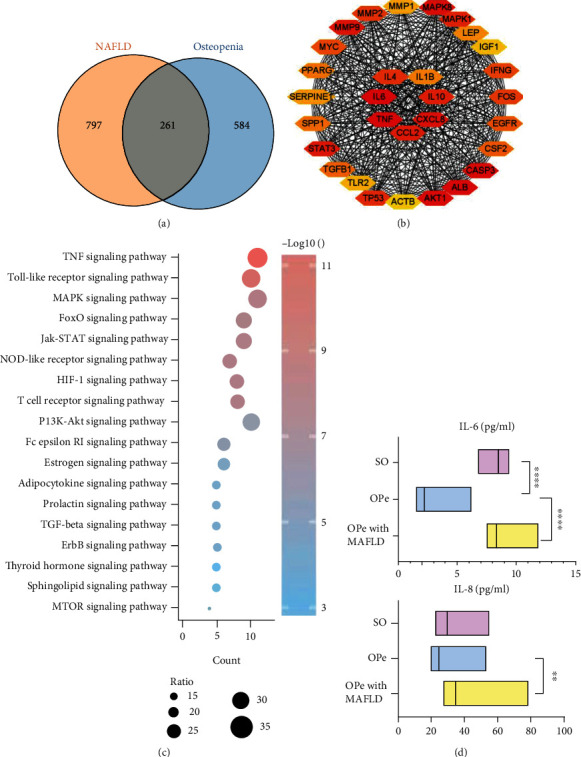
Potential ways of interaction between NAFLD and osteopenia. (a) Venn diagram of the overlap genes between 1058 NAFLD-related genes and 845 osteopenia-related genes from DisGeNET. (b) The hub gene was determined by the construction of PPI network. (c) Top 18 KEGG enrichment pathways of 261overlap genes. (d) The levels of IL-6 and IL-8 in plasma of the patients from SO, OPe, and OPe with MAFLD group were determined by ELISA. Bars, means SE; ∗, *P* < 0.05; ∗∗, *P* < 0.01; ∗∗∗, ∗∗∗∗, *P* < 0.0001. Abbreviations: NAFLD: nonalcoholic fatty liver disease; PPI: protein-protein interaction; KEGG: Kyoto Encyclopedia of Genes and Genomes; ELISA: enzyme-linked immunosorbent assay; IL-6: interleukin-6; SO: severe osteoporosis; OPe: osteopenia; MAFLD: metabolic dysfunction-associated fatty liver disease.

DisGeNET screened out a total of 1058 NAFLD genes and 845 OPe genes which later led to an overlap of 261 targets between NAFLD and OPe by the VENN map (Figure 1(a)). After the construction of the protein-protein interaction (PPI) network, key modules and pivotal hub genes were determined using the STRING and Cytoscape software. This finding suggested that tumor necrosis factor-*α* (TNF-*α*), IL-8, IL-6, IL-4, IL-1*β*, and chemokine (C-C motif) ligand 2 (CCL2) were important pathogenic targets (Figure 1(b)). After that, KEGG pathway annotation showed enrichment of overlapping genes and identification of a total of 111 enriched pathways along with 18 enriched signaling pathways with the highest *p*-adjust values. These pathways included the TNF signal pathway, Toll-like receptor signal pathway, Nod-like receptor signal pathway, hypoxia-inducible factor 1 (HIF-1) signal pathway, and T cell receptor signal pathway (Figure 1(c)). In order to test this hypothesis, the patient's peripheral blood was extracted, and the expression of IL-6 and IL-8 was detected in the elderly OPe patients' plasma with MAFLD, which was remarkably elevated when compared to OPe patients without MAFLD (Figure 1(d)).

**Figure 2:**
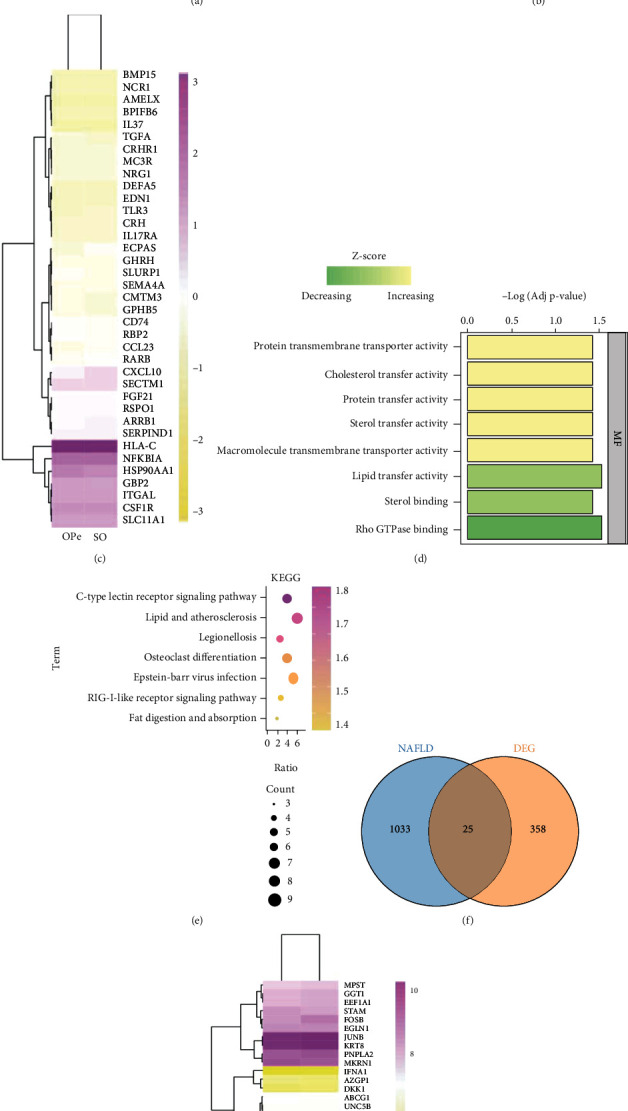
NAFLD and osteopenia may interact through immune pathways. (a) Top 10 GO enrichment items of DEGs between OPe and SO patients from Array Express. (b) Venn diagram of the overlap between immune-related genes and DEGs. (c) The heat map shows the expression of the overlap genes of bone biopsy between OPe and SO patients. (d and e) GO enrichment analysis and KEGG pathway analysis of these 81 overlap genes. (f) Venn plot indicated the overlap between NAFLD-related genes and DEGs. DEGs are derived from the monocytes samples of low and high BMD subjects in GEO database. (g) The heat map indicates the expression level of genes between low and high BMD samples. Abbreviations: SO: severe osteoporosis; OPe: osteopenia; DEG: differentially expressed genes; GO: Gene Oncology; DEGs: differentially expressed genes; KEGG: Kyoto Encyclopedia of Genes and Genomes; BMD: Bone Mineral Density; NAFLD: nonalcoholic fatty liver disease.

The ArrayExpress database extracted one dataset, E-MEXP-1618, which was subsequently made a cohort. The elderly patients over 60 years old were selected as per the received BMD data and divided into the OPe group and the severe osteoporosis (SO) group based on the osteoporosis diagnostic criteria. A total of 13 OPe and 15 SO group samples were analyzed for differentially expressed genes (DEGs) and were subsequently subjected to GO annotations to identify the potential biological functions, as well as differential genes enrichment pathways such as intermembrane lipid transfer and macrophage-derived foam cell differentiation (Figure 2(a)). Additionally, 81 overlapped genes were obtained from 1332 and 1793 genes acquired from DEGs and the Immunology Database and Analysis Portal (ImmPort), respectively (Figure 2(b)), followed by differential gene expression and heat map analysis to determine the gene variation and patterning in bone biopsy between the OPe and SO patients (Figure 2(c)). A subsequent GO and KEGG pathway enrichment analysis on 81 overlapped genes revealed that they were primarily enriched in the lipid metabolism-related pathways (Figures 2(d) and 2(e)). Furthermore, the expression profiling of 73 circulating monocytes by array from 73 pre and postmenopausal females with low or high bone mineral density was downloaded from the GEO database, including 32 low and 41 high BMD group samples, which were analyzed for DEGs, resulting in 25 and 1058 overlapped genes from DEGs and NAFLD from DisGeNET (CUI number: C0400966), respectively (Figure 2(f)). The heat map analysis was also used to ascertain the gene variation in monocytes between the low and high BMD group patients (Figure 2(g)).

### 3.2. Clinical Features, Including the Indicators Related to Muscle Strength and Exercise Performance, Differed between OPe Patients with or without MAFLD

**Figure 3:**
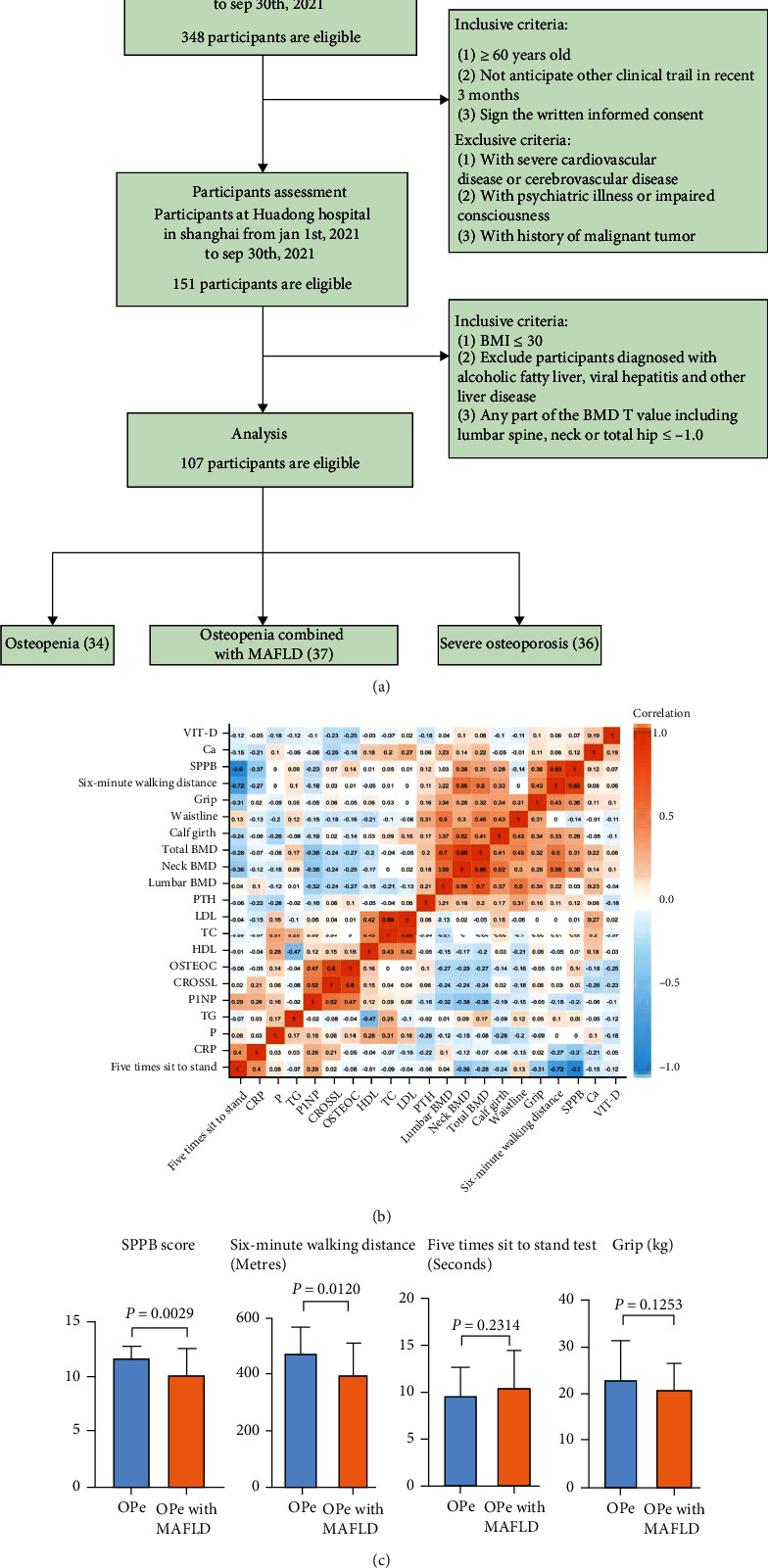
Clinical indicators of all included patients. (a) Flowchart shows the process of subject enrollment and subgroups (OPe grouping =34 subjects, OPe with MAFLD grouping =37 subjects, SO grouping =36 subjects). (b) The correlation matrix of various clinical indicators is presented. (c) Comparison of limb muscle strength in patients between OPe group and OPe with MAFLD group. Abbreviations: SO: severe osteoporosis; OPe: osteopenia; MAFLD: metabolic dysfunction-associated fatty liver disease; SPPB: Short Physical Performance Battery; BMD: bone mineral density; TG: triglycerides; TC: total cholesterol; HDL: high-density lipoprotein; LDL: low-density lipoprotein; P: blood phosphorus; Ca: blood calcium; P1NP: N-Propeptide of Type I Procollagen; CROSSL: *β*-Crosslaps; PTH: parathyroid hormone; OSTEOC: osteocalcin; CRP: C reactive protein.

A flowchart of subject recruitment is shown in Figure 3(a). A total of 107 participants were used in a subsequent analysis, consisting of Group 1: OPe, including osteoporosis patients without MAFLD; Group 2: OPe, including osteoporosis with MAFLD; Group 3: severe osteoporosis without MAFLD (patients with a history of fracture). There was no statistically significant difference in BMI index, blood glucose, comorbidity, smoking and drinking history, sleeping, and nutritional status between Groups 1, 2, and 3 (Table 1).

In order to further observe the effect of MAFLD on muscle strength of elderly patients with OPe, we analyzed the limb muscle strength of Group 1 and Group 2, which was measured by following parameters such as the upper extremities strength was represented by the grip. In contrast, the lower limbs strength was defined by the FTSST and six-minute walking distance, along with the evaluation of lower limb balance by SPPB score. We found that the six-minute walking distance (*P* = 0.012) and SPPB score (*P* = 0.0029) of the elderly OPe patients with MAFLD are worse than those in OPe patients without MAFLD, which suggested that the walking ability and balance ability of the elderly patients with MAFLD are worse (Table 1, Figure 3(c)). Furthermore, after adjusting the confounders, it was observed that the six-minute walking distance reduction was still markedly associated with the prevalence of MAFLD among patients with OPe or osteoporosis (*P* = 0.024) (Table 2).

### 3.3. The Proportion of Peripheral Blood-Derived Macrophages in OPe Patients with or without MAFLD

**Figure 4:**
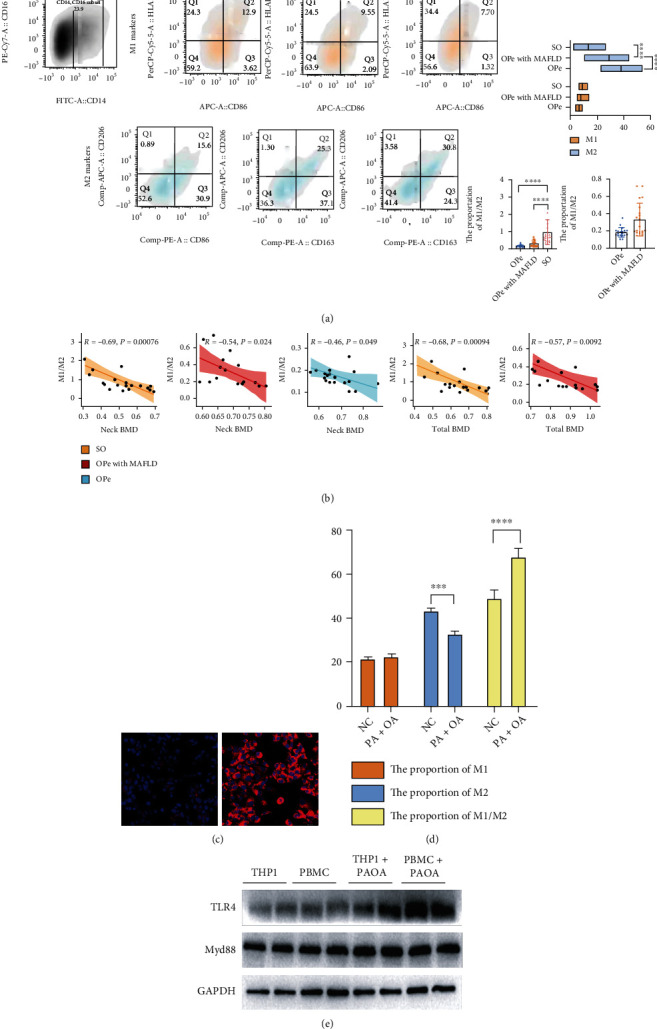
The proportion of peripheral blood-derived macrophages differed between OPe and OPe with MAFLD patients. (a) FCA is performed to compare the M1-like and M2-like monocytes in PBMCs among SO, OPe with MAFLD, and OPe patients. (b) Linear regression analysis showing the relationship between M1-like/M2-like monocytes ratio and bone density-related parameters (neck BMD and total BMD). (c) The validation of fatty liver cell model based on HepG2 cells by Nile red. (d) The proportion of M1 and M2 and M1-M2 ratio is detected by FCA after inducing M0 by conditional media and the supernatant of HepG2 fatty liver model. (e) The protein level of TLR4 and Myd88 in THP-1 induced by PMA and CD14 + momocytes in human peripheral blood are detected by WB. Bars, means SE; ∗, *P* < 0.05; ∗∗, *P* < 0.01; ∗∗∗, *P* < 0.005, ∗∗∗∗, *P* < 0.0001. Abbreviations: FCA: flow cytometric analysis; PBMC: peripheral blood monocytes cell; SO: severe osteoporosis; OPe: osteopenia; MAFLD: metabolic dysfunction-associated fatty liver disease; BMD: bone mineral density; THP-1: human myeloid leukemia mononuclear cells; HepG2: human hepatocellular carcinoma cells; TLR4: toll-like receptor 4; PMA: phorbol 12-myristate 13-acetate; PA + OA: palmitic acid and oleic acid.

In order to investigate the potential role of peripheral blood-derived macrophages in elderly OPe patients with or without MAFLD and SO patients without MAFLD, we first evaluated the levels of macrophages in the peripheral blood of patients using flow cytometric analysis using the gating strategies of macrophage M1-like and M2-like as follows: M1-like (CD14^+^CD16^+^CD86^+^HLA-DR^+^) and M2-like (CD14^+^CD16^+^CD163^+^CD206^+^) (Supplement 1). Our study results demonstrated that the absolute cell counts of M2-like macrophages in PBMCs were significantly increased while M1/M2% was decreased in PBMCs of OPe patients when compared with those with MAFLD and SO patients (Figure 4(a)). Further, an analysis of the correlation between M1/M2% and BMD (neck BMD, lumbar BMD, and total BMD) in all patients revealed that M1/M2% was associated with neck BMD and total BMD, both in OPe with MAFLD group and the SO group patients, thereby suggesting the importance of M1/M2% in the pathogenesis and the progression of OPe in the elderly population (Figure 4(b)). The negative results of the correlation between M1/M2% and BMD are recorded in Supplement 2.

In order to verify the above-mentioned results, a few in vitro experiments were carried out. Firstly, the fatty liver cell model was successfully established by inducing HepG2 cells in vitro with a mixture of FFAs like palmitic and oleic acid (Figure 4(c)), followed by extricating the CD14+ PBMCs extracted from peripheral blood of OPe patients and inducing them to differentiate into M0 macrophages with the fatty liver cell supernatant. The flow cytometry results revealed that the decrease in M2% and the increase in M1/M2% proportion suggested that the intervention of fatty liver cell supernatant can promote differentiation of M0 macrophages into M1-like macrophages (Figure 4(d)). The WB results also revealed that the protein expression levels of toll-like receptor 4 (TLR4) and myeloid differentiation factor 88 (MyD88), a class of important protein molecules involved in innate immunity, increased significantly following the intervention in both THP-1 cells and CD14+ PBMCs (Figure 4(e)).

### 3.4. MAFLD Impairs CD14+ Mononuclear Cellular Aerobic Respiration and Mitochondrial Homeostasis in Elderly OPe Patients

**Figure 5:**
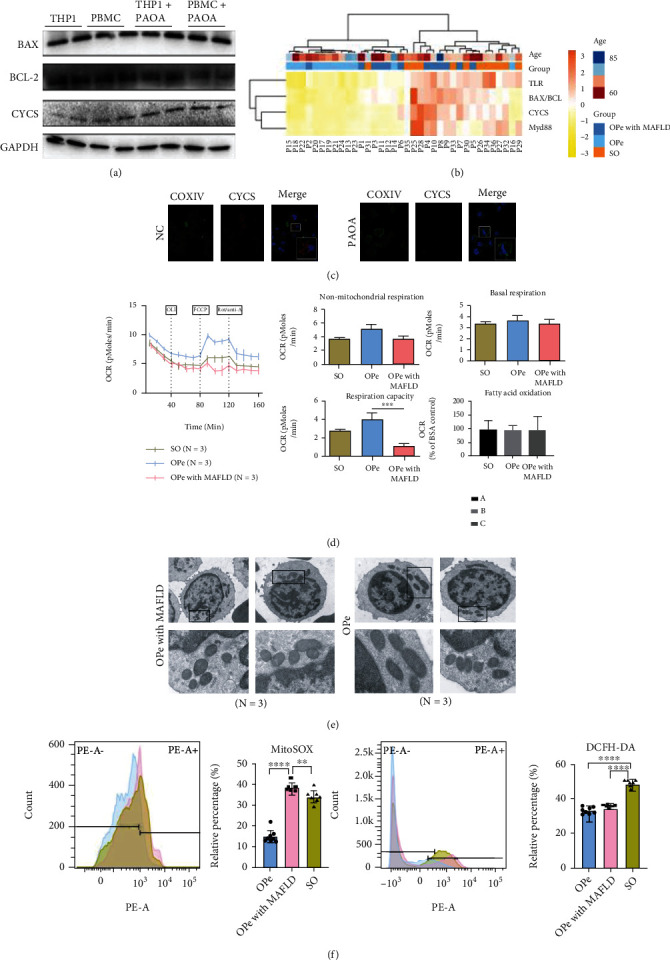
MAFLD impaired aerobic respiration and mitochondrial morphology of CD14+ monocytes in peripheral blood of patients with osteopenia. (a) The protein level of BAX, BLC2, and CYCS in Thp-1 induced by PMA and CD14 + momocytes in human peripheral blood is detected by WB. (b) In the heat map, the relative mRNA level of TLR4, BAX/BCL-2, CYCS, and Myd88 in PBMCs from OPe, OPe with MAFLD, and SO patients is determined by RT-PCR. (c) Representative image from immunofluorescence staining assay of COXIV and CYCS in THP-1 induced by PMA and the supernatant of HepG2 fatty liver model. COXIV (green), CYCS (red). (d) The OCR and ECAR in CD14 + PBMCs from the patients among OPe, OPe with MAFLD, and SO are detected by Seahorse XF. The bar plots indicate the quantitation of non-mitochondrial respiration, basal respiration, respiration capacity, and fatty acid oxidation among OPe, OPe with MAFLD, and SO. (e) The mitochondrial microstructure and morphology of CD14+ PBMCs from OPe and OPe with MAFLD patients are observed via transmission electron microscopy. (f) The level of mitochondrial ROS and intracellular ROS is assessed by FCA among OPe, OPe with MAFLD, and SO. The quantitative results showed right. Bars, means SE; ∗, *P* < 0.05; ∗∗, *P* < 0.01; ∗∗∗, *P* < 0.005; ∗∗∗∗, *P* < 0.0001. Abbreviations: TLR4: toll-like receptor 4; MyD88: myeloid differentiation factor 88; BCL-2: B-cell lymphoma-2; BAX: BCL2-associated X protein; CYCS: Cytochrome C; COXIV: Cytochrome c oxidase subunit IV; ROS: reactive oxygen species; PBMC: peripheral blood monocytes cell; SO: severe osteoporosis; OPe: osteopenia; OPe with MAFLD: osteopenia with metabolic dysfunction-associated fatty liver disease.

WB results displayed that the BCL2-associated X protein (BAX) and Cytochrome C (CYCS) genes expression levels increased with THP-1 and PBMCs extracted from OPe patients after the HepG2 supernatant PAOA intervention, while the expression of B-cell lymphoma-2 (BCL-2) was decreased (Figure 5(a)). Furthermore, the heat map analysis revealed that the relative BAX/BCL-2, CYCS, TLR4, and MyD88 mRNA expressions in PBMCs from OPe patients with MAFLD group and SO group patients were elevated as compared to OPe patients (Figure 5(b)), which were further confirmed by IF staining that observed CYCS upregulation in THP-1 cells after the intervention (Figure 5(c)).

Owing to the fact that mitochondria, as highly dynamic organelles, can adjust their morphology depending on the energy demand and metabolic conditions in the majority of cells, the CD14+ PBMCs bioenergetics analysis used the Seahorse XF Extracellular Flux Analyzer, which simultaneously quantifies two energetic pathways-glycolysis denoted by ECAR and oxidative phosphorylation measured by OCR, suggesting that CD14+ PBMCs from OPe patients with MAFLD group consumed oxygen at a lower basal level and produced less adenosine 5-triphosphate (ATP), when compared to patients in OPe and SO groups, and showed the least OCR increase in response to FCCP, resulting in a significant decrease in maximum respiratory capacity (Figure 5(d)) followed by the analysis of the mitochondria's state in CD14+ monocytes by TEM that indicated severe mitochondrial dysfunction developed in the CD14+ monocytes in MAFLD patients along with swelling up of mitochondrial cristae (Figure 5(e)). The oxidative stress was assessed by intracellular ROS quantification, especially mtROS (Figure 5(f)), exhibiting that the mtROS level in CD14+ PBMCs in OPe patients with the MAFLD group was the highest among all.

**Figure 6:**
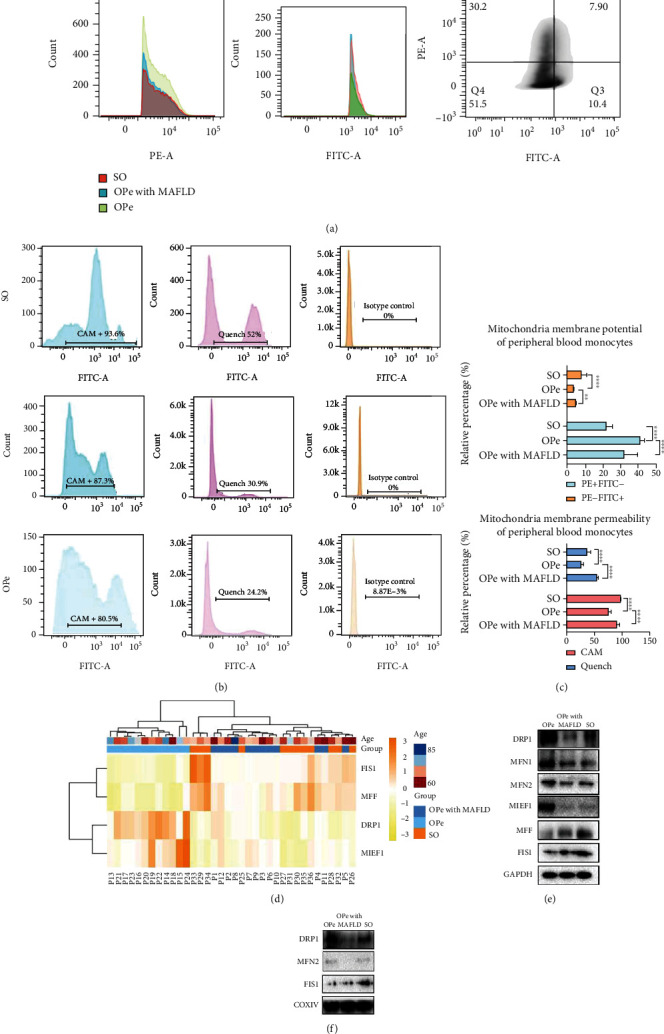
MAFLD impairs the function of CD14 + mononuclear mitochondria in peripheral blood of osteopenia patients. (a, b) MMP and MPTP of PBMCs from the patients among OPe, OPe with MAFLD, and SO based on FCA. (c) The quantitative results of MMP and MPTP are, respectively, presented in bar plots. (d) In the heat map, the relative mRNA level of FIS1, MFF, DRP1, and MIEF1 in PBMCs from the patients among OPe, OPe with MAFLD, and SO group is determined by RT-PCR. (e) The protein level of mitochondrial fusion and division-related protein in the PBMCs from patients among OPe, OPe with MAFLD, and SO group detected by WB. (f) The protein level of mitochondrial fusion and division-related protein in the mitochondrial of PBMCs from patients among OPe, OPe with MAFLD, and SO group detected by WB. Bars, means SE; ∗, *P* < 0.05; ∗∗, *P* < 0.01; ∗∗∗, *P* < 0.005; ∗∗∗∗, *P* < 0.0001. Abbreviations: MMP: mitochondrial membrane potential; MPTP: mitochondrial permeability transition pore; PBMC: peripheral blood monocytes cell; SO: severe osteoporosis; OPe: osteopenia; OPe with MAFLD: osteopenia with metabolic dysfunction-associated fatty liver disease; DRP1: dynamin-related protein 1; FIS1: mitochondrial fission 1 protein; MFF: mitochondrial fission factor; MIEF1: mitochondrial elongation factor 1; MFN1: mitofusin 1; MFN2: mitofusin 2; COXIV: Cytochrome c oxidase subunit IV.

Mitochondrial function was also assessed by the MMP and MPTP using flow cytometry detection analysis, which revealed that the mitochondrial membrane potential of PBMCs in OPe patients with MAFLD was lower while the membrane permeability was higher than the OPe group without MAFLD (Figures 6(a)–6(c)). The protein and gene expressions of fission proteins like dynamin-related protein 1 (DRP1), mitochondrial fission 1 protein (FIS1), mitochondrial fission factor (MFF), a mitochondrial dynamic fusion protein, and mitochondrial elongation factor 1 (MIEF1) were evaluated by RT-PCR and WB. The data revealed decreased DRP1 and MIEF1 levels, with a concurrent increase in MFF and FIS1 levels in CD14+ PBMCs in OPe patients with MAFLD and SO patients compared to the OPe group (Figures 6(d) and 6(e)). A subsequent WB test on extracted mitochondria from CD14+ PBMCs from all groups revealed that, while there was no difference in FIS1 expression in PBMC mitochondria in both OPe and OPe with MAFLD patients, DRP1 and mitofusin 2 (MFN2) expression levels decreased significantly (Figure 6(f)).

### 3.5. SIRT1 Defects Accentuate Impaired Mitochondrial Monocytes in the Elderly Osteopenia Patients

**Figure 7:**
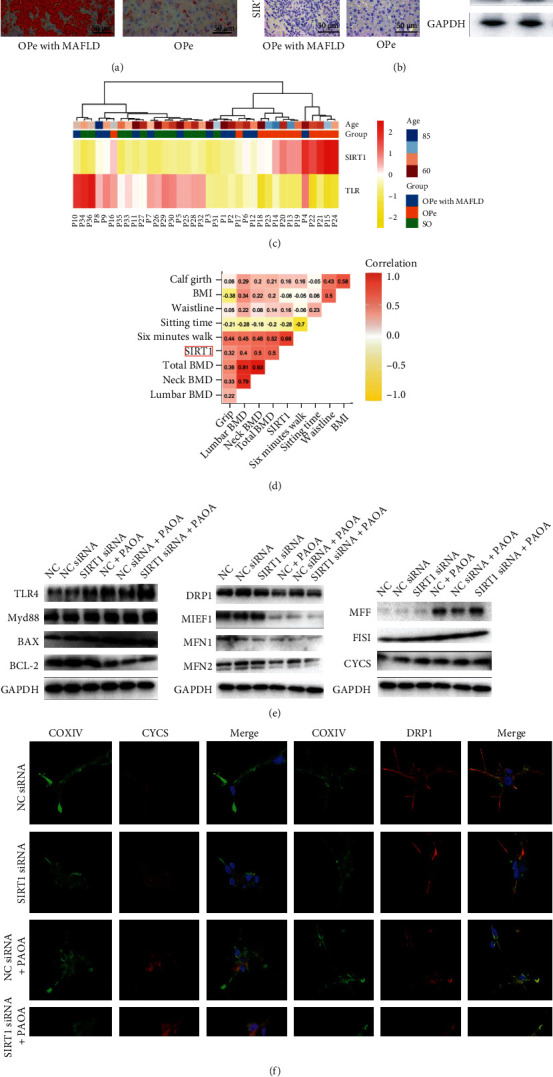
SIRT1 defects accentuated impaired monocyte mitochondria in osteopenia patients. (a) The accumulation of lipid droplets of liver from OPe and OPe with MAFLD patients is assessed by Red Oil O staining. (b) Immunohistochemical detection of SIRT1 in the liver from OPe and OPe with MAFLD patients. The protein level of SIRT1 in liver tissue is assessed by WB. (c) The relative mRNA level of SIRT1 and TLR4 in PBMCs from the patients among OPe, OPe with MAFLD, and SO group is detected by RT-PCR. (d) The correlation matrix shows the relationship between the expression of SIRT1 and clinical indicators of patients. (e) The protein level of mitochondrial apoptosis, fusion and division-related protein is detected using WB. (f) Representative image for immunofluorescence staining shows the expression and distribution of COXIV and CYCS in PBMCs intervened by various conditioned medium. Abbreviations: SIRT1: the silent mating type information regulation 1; TLR4: toll-like receptor 4; MyD88: myeloid differentiation factor 88; BCL-2: B-cell lymphoma-2; BAX: BCL2-associated X protein; CYCS: Cytochrome C; DRP1: dynamin-related protein 1; FIS1: mitochondrial fission 1 protein; MFF: mitochondrial fission factor; MIEF1: mitochondrial elongation factor 1; MFN1: mitofusin 1; MFN2: mitofusin 2; COXIV: Cytochrome c oxidase subunit IV; PAOA: palmitic acid and oleic acid; BMI: body mass index; BMD: bone mineral density; SO: severe osteoporosis; OPe: osteopenia; OPe with MAFLD: osteopenia with metabolic dysfunction-associated fatty liver disease.

The SIRT1 expression level in OPe patients with MAFLD and without MAFLD was evaluated by IHC and WB to investigate the role of SIRT1 (Figure 7(a)). It was observed that SIRT1 expression level was reduced in liver tissue from MAFLD patients (Figure 7(b)). An RT-PCR assessment of mRNA expression profile in PBMCs obtained from all included patients showed that patients in OPe with MAFLD and SO group were clustered into one group according to the system cluster and hierarchical cluster analysis. The relative SIRT1 mRNA expression in PBMCs from OPe patients was significantly higher than that in OPe with MAFLD group and SO group (Figure 7(c)). The heat map was also utilized to exhibit the associations between the SIRT1 expression and various clinical indices such as BMD, limb muscle strength assessment, waistline, calf girth in three group patients, which suggested that the SIRT1 level was highly correlated with neck BMD (*r* = 0.5), total BMD (*r* = 0.5) and six-minute walking distance (*r* = 0.68) (Figure 7(d)).

To investigate the role of SIRT1 in macrophage mitochondrial function further, CD14+ PBMCs extracted from OPe patients were treated with the supernatant from HepG2 cells treated in vitro with PAOA. Meanwhile, a decreased SIRT1 expression in CD14+ PBMCs by siRNA was used before the stimulation of PAOA, which observed that the DRP1, MIEF1, MFN1, MFN2 (mitochondrial fusion proteins), and BCL-2 (mitochondrial anti-apoptotic protein) levels were decreased after the intervention, whereas the MFF, FIS1 (mitochondrial fission proteins) along with CYCS and BAX (mitochondrial apoptosis-related proteins) levels were upregulated in WB results (Figure 7(e)) and were further substantiated by IF that DRP1 downregulation and CYCS upregulation after the intervention that might be due to downregulation of SIRT1 expression by siRNA (Figure 7(f)). Moreover, TLR4 and MyD88 protein levels also increased after intervention, thereby indicating that SIRT1 expression is crucial for modulating the mitochondrial function of peripheral blood-derived macrophages in OPe patients (Figure 7(e)).

## 4. Discussion

Metabolic diseases have always been thought to be a significant risk factor for osteoporosis, whereas fatty liver disease is usually associated with metabolic dysfunction and is a major predisposing factor in obesity and diabetes, leading to chronic inflammation [30, 31]. Our study results revealed weaker walking ability in OPe patients with MAFLD than those without MAFLD. Several studies have proposed that pro-inflammatory cytokines secreted after macrophage polarization might contribute to skeletal muscle aging [32]. Oxidative stress is an important mechanism of osteoporosis. Free radicals affect the differentiation, function, and apoptosis of osteoblasts and osteoclasts by regulating signal pathways or inducing inflammatory reactions. Antioxidants can effectively prevent and treat osteoporosis [33]. Initial research, led by Narayan Avadhani of the University of Pennsylvania, concluded that when mitochondrial function is affected, it will not only affect energy production, but also trigger a stress signal that induces excessive production of osteoclasts. In the future, they will study how to prevent osteoporosis by protecting mitochondrial function [34]. Furthermore, recent studies indicate that mitochondrial regulators/nutrients from natural products can remedy mitochondrial dysfunction mediated by MAFLD [35, 36]. Meanwhile, a number of researches nowadays are developing innovative drugs for the prevention and treatment of fatty liver diseases based on mitochondrial dysfunction [37]. The role of mitochondria as the metabolic center of cells in regulating macrophage function has been gradually revealed [38]. This study attempts to clarify whether MAFLD reduces the walking ability of patients by affecting the mitochondrial function of peripheral blood-derived macrophages in the elderly OPe patients.

Fragility fracture is a complete fracture caused by a spontaneous or slight external force, which is the most serious consequence of osteoporosis [39]. In our study, all participants were divided into three groups: OPe (including osteoporosis) patients with MAFLD (OPe with MAFLD), OPe patients (including osteoporosis) without MAFLD, and SO patients without MAFLD who experienced fragility fractures. Activation of the monocyte-macrophage system is an important feature of chronic inflammation, which was found by the flow cytometry that the M1/M2 ratio in PBMCs in SO patients was the highest among the three groups. Further analysis of the OPe patients with and without MAFLD denoted that the M1/M2 ratio in the MAFLD group was higher than that in the group without MAFLD, although it was not statistically significant. TLR4 is mainly expressed in cells involved in host defense function, such as monocyte-macrophages, dendritic cells, and lymphocytes, which mediate chronic inflammation [12], along with an essential adapter protein, MyD88, which is crucial for all TLRs except TLR3 in the innate immunity system [40]; our results displayed that the TLR4 and MyD88 expressions in PBMCs obtained from MAFLD patients were significantly higher than those without MAFLD.

The cells of the innate immune system, including macrophages and antigen-presenting cells, play a vital role in providing host resistance to infection and promoting inflammatory response [41], which were also corroborated by various studies that the mitochondrial morphological changes were involved in the regulation of cellular metabolism, which may indirectly affect the activation and response of immune cells [42, 43] along with the presence of mtROS, produced by electron transport chain (ETC) can trigger innate immune signals or cause immune cell damage in accordance with the measure and timing of their production [44]. Chronic inflammation results in the release of a substantial number of cellular mtROS into the blood, thereby interfering with their functions and disrupting intercellular communication [45]. Although ROS levels were comparable in patients with and without MAFLD, mtROS levels in peripheral blood of OPe patients with MAFLD were significantly higher than those without MAFLD. It was also evident that JC-1, mitochondrial permeability test, TEM, and WB tests also stated that peripheral blood macrophages in OPe patients with MAFLD possessed more damaged mitochondria than other groups.

Mitochondrial oxidative phosphorylation (OXPHOS) provides sufficient energy to perform all cellular tasks through aerobic metabolism, which converts energy substrate into energy stored in the ATP. Although OXPHOS carries out electron transfer in the mitochondrial membrane respiratory chain to produce ATP [46], inflammatory macrophages sometimes enhance glycolytic metabolism and inhibit mitochondrial OXPHOS [47]. The present study employed seahorse XF Analyzer to detect oxidative phosphorylation and glycolysis of CD14^+^ PBMCs obtained from all three groups and suggested that the monocyte respiratory capacity in patients with OPe with MAFLD is the lowest among all, even when compared to SO patients.

Mitochondria are complex dynamic organelles that perform many functions related to cell metabolism and homogeneous stability [48] and bear a close association with the process of aging [49] that might reduce mitochondrial integrity as well as dysfunction of the fusion-fission cycle, resulting in the accumulation of a large number of abnormal mitochondria, leading to an increase in oxidative stress and resulting in defective autophagy [50]. As the center of cell energy metabolism, the mitochondrial shape constantly changes through different fusion and fission cycles to adapt to the varied energy needs of the different environments [51]. Mitochondrial key proteins that induce fusion and fission and dynamic proteins play critical regulatory roles in the process of constitutive fission and fusion reactions, which maintain steady-state mitochondrial morphology [52]. The present study assessed the relative mRNA expression and protein level of genes related to mitochondrial fusion-fission cycle in PBMCs of all the three group patients by RT-PCR and WB, and proved that the expression levels of mitochondrial fusion and division-related proteins was abnormal in PBMCs obtained from patients with MAFLD and SO patients when compared with patients without MAFLD.

As a regulator of various cellular and body processes, including metabolism, immune response, and aging, SIRT1 remains the most studied member of this class of proteins engaging in gene regulation [53]. SIRT1, a NAD-dependent histone deacetylase, plays a vital role in hepatic steatosis and inflammation [54], along with active participation in other cellular events like metabolism, inflammatory response, cell aging, and apoptosis through a variety of signaling pathways. In our study, it was reflected that the SIRT1expression was lesser in the liver tissue of OPe patients with MAFLD than patients without MAFLD. In order to further clarify the role of SIRT1, we observed the effects of SIRT1 siRNA on PBMCs obtained from OPe patients on cell signaling and the mitochondrial function of cells by WB and IF, which suggested that SIRT1 knockdown aggravated the mitochondrial damage of induced monocytes in vitro, which might be related to the TLR4 signal activation.

“Inflammatory aging” is the most common manifestation of abnormal intercellular communication. Age-related dysfunction and immune system decline can stimulate a large number of immune cells to produce numerous inflammatory factors and cause chronic inflammation, potentially accelerating the aging process. Our study revealed that OPe patients with MAFLD had increased levels of plasma pro-inflammatory factors like IL-6 and IL-8 and higher M1/M2% as compared to the OPe patients without MAFLD. To conclude, our results suggest that MAFLD itself may aggravate the inflammatory state of the elderly OPe patients, which may be related to the mitochondrial homeostasis imbalance in peripheral blood-derived macrophages that might lead to the decreased walking ability of patients.

## Figures and Tables

**Table 1:** Baseline characteristics and clinical features.

	OPe	OPe with MAFLD	SO	*P*-value
Gender	12/22	12/25	3/33	0.016
Age	67.88 ± 5.05	67.32 ± 6.15	70.69 ± 7.54	0.070
BMI	24.42 ± 2.05	25.42 ± 2.29	22.28 ± 2.40	0.648
Smoke	26/4	32/5	29/7	0.724
Alcohol	25/5	32/5	30/6	0.914
Hypertension	18/11	22/15	26/10	0.490
Diabetes	28/1	31/6	30/6	0.207
Exercise (yes or no)	9/17	23/11	18/13	0.035
MNA	12.66 ± 1.60	12.86 ± 1.78	10.89 ± 2.27	0.116
PSQI	6.55 ± 4.95	8.03 ± 4.56	9.69 ± 5.23	0.733
Calf girth	33.44 ± 2.87	33.73 ± 3.67	30.47 ± 3.01	0.293
Waist line	84.03 ± 8.12	87.38 ± 8.10	80.47 ± 7.95	0.991
FTSST	9.59 ± 3.04	10.62 ± 3.91	14.62 ± 7.70	<0.001
Six-minute walking	470.73 ± 98.44	405.51 ± 107.34	326.41 ± 112.62	0.755
SPPB	11.56 ± 1.24	10.06 ± 2.50	8.06 ± 3.05	<0.001
Grip	22.08 ± 7.55	19.68 ± 5.25	17.73 ± 5.03	0.034
Ca	2.29 ± 0.11	2.29 ± 0.087	2.25 ± 0.08	0.309
P	1.11 ± 0.13	1.11 ± 0.16	1.14 ± 0.15	0.542
P1NP/CROSSL	0.10 ± 0.03	0.11 ± 0.06	0.14 ± 0.17	<0.001
CROSSL	500.71 ± 227.88	499.88 ± 248.11	610.19 ± 322.61	0.146
PTH	43.09 ± 15.21	47.43 ± 13.42	42.51 ± 17.57	0.338
VITAMIN D	21.90 ± 9.60	17.82 ± 5.91	16.86 ± 7.29	0.036
OSTEOC	19.01 ± 7.68	17.15 ± 5.91	19.58 ± 6.50	0.360
P1NP	46.44 ± 14.25	46.48 ± 19.58	60.93 ± 34.67	<0.001
Lumbar BMD	0.90 ± 0.15	0.92 ± 0.16	0.74 ± 0.15	0.889
Neck BMD	0.67 ± 0.08	0.65 ± 0.08	0.47 ± 0.19	<0.001
Total BMD	0.81 ± 0.10	0.82 ± 0.10	0.61 ± 0.12	0.711
ALT	16.56 ± 7.63	20.52 ± 11.13	15.43 ± 8.85	0.096
AST	18.77 ± 4.88	20.79 ± 7.77	18.18 ± 5.65	0.023
TC	4.76 ± 0.98	4.74 ± 0.99	4.48 ± 0.90	0.824
TG	1.37 ± 0.41	2.09 ± 1.38	1.53 ± 0.94	<0.001
LDL	2.87 ± 0.90	2.71 ± 0.85	2.52 ± 0.80	0.849
HDL	1.48 ± 0.28	1.37 ± 0.38	1.48 ± 0.29	0.209
BUN	5.58 ± 1.34	5.30 ± 1.28	5.67 ± 1.41	0.840
Scr	70.64 ± 14.72	69.87 ± 15.62	68.71 ± 18.38	0.010
SUA	311.55 ± 63.42	338.03 ± 60.47	276.41 ± 76.99	0.324
eGFR	84.12 ± 12.23	83.89 ± 11.12	83.86 ± 16.93	0.037
Lumbar-t value	−1.18 ± 1.08	-1.05 ± 1.24	-2.51 ± 1.28	0.564
Neck of femur-t value	−1.64 ± 0.59	−1.87 ± 0.66	−3.24 ± 0.87	0.064
Total-t value	−1.38 ± 0.75	−1.23 ± 0.82	−2.91 ± 1.04	0.153

*Abbreviations*: OPe: osteopenia; OPe with MAFLD: osteopenia combined with MAFLD; SO: severe osteoporosis; BMI: body mass index; FTSST: five times sit to stand test; PSQI: Pittsburgh Sleep Quality Index; MNA-SF: Mini Nutritional Assessment-Short Form; SPPB: Short Physical Performance Battery; BMD: bone mineral density; eGFR: Estimated Glomerular Filtration Rate; TG: triglycerides; TC: total cholesterol; Scr: serum creatinine; SUA: serum uric acid; BUN: blood urea nitrogen; HDL: high-density lipoprotein; LDL: low-density lipoprotein; ALT: alanine aminotransferase; AST: aspartate transaminase; P: blood phosphorus; Ca: blood calcium; P1NP: N-Propeptide of Type I Procollagen; CROSSL: *β*-Crosslaps; PTH: parathyroid hormone; OSTEOC: osteocalcin. The *P* value in red means that the *P* value <0.05 and is considered to be significantly important.

**Table 2:** Multiple logistic regression analysis of muscle strength related indexes in elderly patients with osteopenia (including osteoporosis) with or without MAFLD.

	OR	95% CI	*P*-value
Exercise	1.64	0.20–13.67	0.648
FTSST	1.44	–0.96–2.16	0.082
Six-minute walking	1.02	1.00–1.04	0.024
Grip	1.19	0.94–1.51	0.140
P1NP/CROSSL	714.54	8.90e − 10–5.74e + 14	0.638
VITMIN D	1.10	0.98–1.23	0.122
P1NP	1.03	0.97–1.09	0.380
AST	0.96	0.82–1.12	0.590
TG	0.13	0.02–1.09	0.059
Scr	0.88	0.75–1.03	0.121
eGFR	0.93	0.78–1.10	0.375

*Abbreviations*: OPe: osteopenia; OPe with MAFLD: osteopenia combined with MAFLD; SO: severe osteoporosis; FTSST: five times sit to stand test; eGFR: Estimated Glomerular Filtration Rate; Scr: serum creatinine; TG: triglycerides; AST: aspartate transaminase; P1NP: N-Propeptide of Type I Procollagen; CROSSL: *β*-Crosslaps. The *P* value in red means that the *P* value <0.05 and is considered to be significantly important.

## Data Availability

The data used to support the findings of this study are available from the corresponding authors upon request.

## Abbreviations

MAFLD: Metabolic dysfunction-associated fatty liver disease
NAFLD: Nonalcoholic fatty liver disease
FLD: Fatty liver disease
OPe: Osteopenia
SO: Severe osteoporosis
T2DM: Type 2 diabetes mellitus
BMD: Bone mineral density
WHO: World Health Organization
PSQI: Pittsburgh sleep quality index
MNA-SF: Mini nutritional assessment-short form
SPPB: Short physical performance battery
FTSST: Five times sit to stand test
BW: Body weight
BH: Body height
BMI: Body mass index
eGFR: Estimated Glomerular Filtration Rate
TG: Triglycerides
TC: Total cholesterol
Scr: Serum creatinine
SUA: Serum uric acid
BUN: Blood urea nitrogen
HDL: High-density lipoprotein
LDL: Low-density lipoprotein
ALT: Alanine aminotransferase
AST: Aspartate transaminase
P: Blood phosphorus
Ca: Blood calcium
P1NP: N-Propeptide of Type I Procollagen
CROSSL: *β*-Crosslaps
PTH: Parathyroid hormone
OSTEOC: Osteocalcin
CRP: C reactive protein
KEGG: Kyoto Encyclopedia of Genes and Genomes
GEO: Gene Expression Omnibus
GO: Gene Oncology
DisGeNET: Gene disease database
PPI: Protein-protein interaction
DEGs: Differentially expressed genes
ImmPort: The Immunology Database and Analysis Portal
THP-1: Human myeloid leukemia mononuclear cells
PBMC: Peripheral blood mononuclear cells
HepG2: Human hepatocellular carcinoma cells
DMEM: Dulbecco's modified eagle medium
FBS: Fetal bovine serum
RT: Room temperature
BSA: Bovine serum albumin
PBS: Phosphate buffer saline
BCA: Bicinchoninic acid
FFAs: Free fatty acids
PFA: Paraformaldehyde
PAOA: Palmitic acid and oleic acid
FCCP: Carbonyl cyanide p-(trifluoromethoxy)phenylhydrazone
2-DG: 2-deoxy-D-glucose
TEM: Transmission electron microscopy
FCM: Flow cytometry
ROS: Reactive oxygen species
mtROS: Mitochondrial reactive oxygen species
MPTP: Mitochondrial permeability transition pore
MMP: Mitochondrial membrane potential
ECAR: Extracellular acidification rate
OCR: Oxygen consumption rate
WB: Western blotting
IHC: Immunohistochemistry
IF: Immunofluorescence
ELISA: Enzyme-linked immunosorbent assay
IL-6: Interleukin-6
TNF-*α*: Tumor necrosis factor-*α*
CCL2: Chemokine (C-C motif) ligand 2
HIF-1: Hypoxia-inducible factor 1
SIRT1: The silent mating type information regulation 1
TLR4: Toll-like receptor 4
MyD88: Myeloid differentiation factor 88
BCL-2: B-cell lymphoma-2
BAX: BCL2-associated X protein
CYCS: Cytochrome C
DRP1: Dynamin-related protein 1
FIS1: Mitochondrial fission 1 protein
MFF: Mitochondrial fission factor
MIEF1: Mitochondrial elongation factor 1
MFN1: Mitofusin 1
MFN2: Mitofusin 2
COXIV: Cytochrome c oxidase subunit IV
siRNA: Small interfering RNA
ETC: Electron transport chain
OXPHOS: Oxidative phosphorylation
ATP: Adenosine 5-triphosphate.

## Supplementary Materials

Supplementary Materials Supplement 1. The process of Macrophage flowchart. Supplement 2. Negative results of correlation between M1/M2% and bone mineral density. Supplement 3. The sequences of all the primers used. Supplement 4. All IF and WB antibodies used in this study.
