# Supplementary material for: The Imbalance of Mitochondrial Homeostasis of Peripheral Blood-Derived Macrophages Mediated by MAFLD May Impair the Walking Ability of Elderly Patients with Osteopenia
Source: Oxid Med Cell Longev. 2022 Mar 24;2022:5210870. doi: 10.1155/2022/5210870 (PMC8970807; doi:10.1155/2022/5210870)
Supplement: Supplementary Materials — Supplement 1. The process of Macrophage flowchart. Supplement 2. Negative results of correlation between M1/M2% and bone mineral density. Supplement 3. The sequences of all the primers used. Supplement 4. All IF and WB antibodies used in this study. [file 5210870.f1.docx]

**Supplements:**

**Supplement 1. The process of Macrophage flowchart.**

**
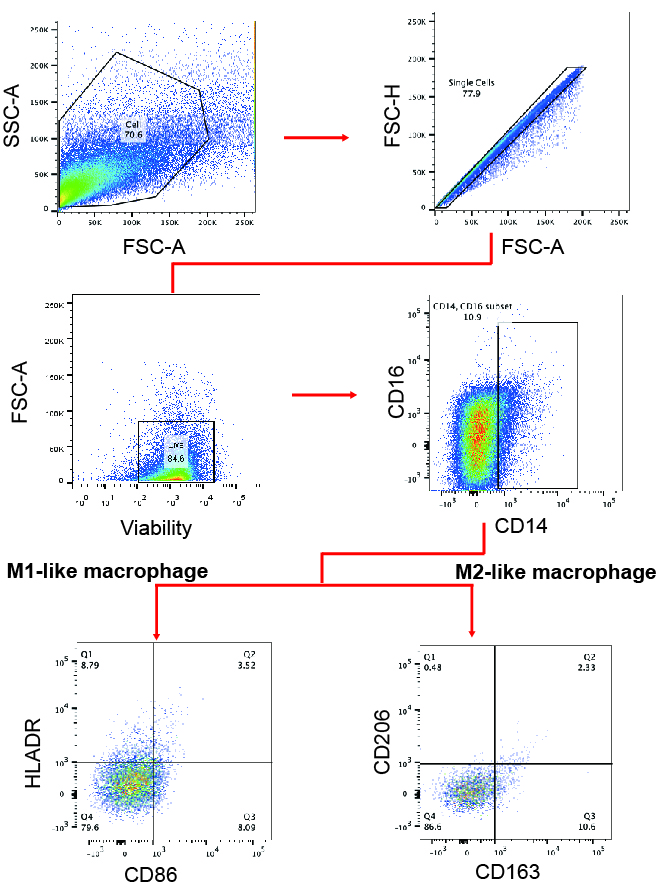
**

**Supplement 2. Negative results of correlation between M1/M2% and bone mineral density**

**
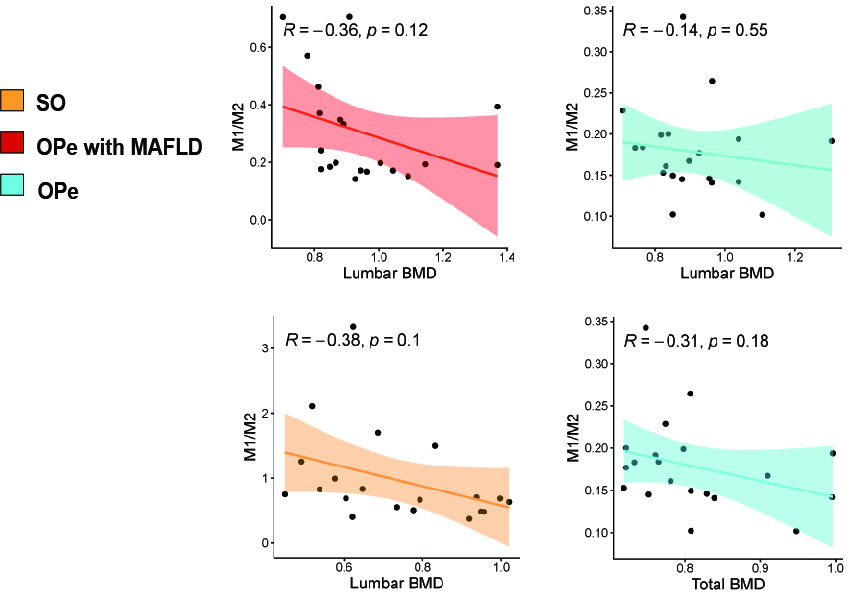
**

***Abbreviations***: OPe, Osteopenia; OPe with MAFLD, Osteopenia combined with MAFLD; BMD, Bone Mineral Density.

**Supplement 3. The sequences of all the primers used.**

| Primer | Forward | Reverse |
| --- | --- | --- |
| TLR4 | CTTGGTGGAAGTTGAACGAATGGAATG | CAAGCACACTGAGGACCGACAC |
| BAX | AGCGACTGATGTCCCTGTCTCC | AGATGGTGAGTGAGGCGGTGAG |
| BCL-2 | TCGCCCTGTGGATGACTGAGTAC | TCAGAGACAGCCAGGAGAAATCAAAC |
| CYCS | AAAGGGAGGCAAGCACAAGACTG | TTATTGGCGGCTGTGTAAGAGTATCC |
| Myd88 | GCCGCCTGTCTCTGTTCTTGAAC | GGTCCGCTTGTGTCTCCAGTTG |
| SIRT1 | TTCTTGTGGCAGTAACAGTGATAGTGG | CTCTGGAACATCAGGCTCATCTTCTAAG |
| GAPDH | CCTTCCGTGTCCCCACT | GCCTGCTTCACCACCTTC |

**Supplement 4. All IF and WB antibodies used in this study**

| Antibodies | Source | Identifier |
| --- | --- | --- |
| BAX Rabbit pAb | ABclonal | A19684 |
| BCL-2 Rabbit pAb | ABclonal | A0208 |
| Cytochrome c Rabbit pAb | ABclonal | A13430 |
| TLR4 Rabbit pAb | ABclonal | A11226 |
| Myd88 Rabbit pAb | ABclonal | A0786 |
| SIRT1 Rabbit Polyclonal Antibody | Proteintech | 13161-1-AP |
| DRP1 Rabbit pAb | ABclonal | A17069 |
| MIEF1 Rabbit pAb | ABclonal | A10396 |
| MFN1 Rabbit pAb | ABclonal | A9880 |
| MFN2 Rabbit pAb | ABclonal | A12771 |
| MFF Rabbit pAb | ABclonal | A12392 |
| TTC11/FIS1 Rabbit mAb | ABclonal | A19666 |
| COXIV Rabbit pAb | ABclonal | A6564 |
| GAPDH Rabbit pAb | ABclonal | A19056 |
